# The genome of the Australian water dragon (*Intellagama lesueurii*), an agamid model for urban adaptation

**DOI:** 10.1093/jhered/esae054

**Published:** 2024-10-04

**Authors:** Daniel Powell, Nicola Jackson, Parwinder Kaur, Olga Dudchenko, Erez Lieberman Aiden, Arthur Georges, Céline Henria Frère

**Affiliations:** School of the Environment, Faculty of Science, University of Queensland, St Lucia, QLD, Australia; Centre for Bioinnovation, University of the Sunshine Coast, Sippy Downs, QLD, Australia; School of the Environment, Faculty of Science, University of Queensland, St Lucia, QLD, Australia; UWA School of Agriculture and Environment, The University of Western Australia, Perth, WA, Australia; Department of Molecular and Human Genetics, The Center for Genome Architecture, Baylor College of Medicine, Houston, TX, United States; The Center for Theoretical Biological Physics, Rice University, Houston, TX, United States; Department of Molecular and Human Genetics, The Center for Genome Architecture, Baylor College of Medicine, Houston, TX, United States; The Center for Theoretical Biological Physics, Rice University, Houston, TX, United States; Broad Institute of MIT and Harvard, Cambridge, MA, United States; Institute for Applied Ecology, University of Canberra ACT 2601, Australia; School of the Environment, Faculty of Science, University of Queensland, St Lucia, QLD, Australia

**Keywords:** chromosome-length, comparative analysis, *Intellagama*, reptile genome, water dragon

## Abstract

Squamate reptiles are a highly diverse and intriguing group of tetrapods, offering valuable insights into the evolution of amniotes. The Australian water dragon (*Intellagama lesueurii*) is a member of the Agamidae and sister to the core mesic Australian endemic radiation (Amphibolurinae). The species is renowned for its urban adaptability and complex social systems. We report a 1.8 Gb chromosome-length genome assembly together with the annotation of 23,675 protein-coding genes. Comparative analysis with other squamate genomes highlights gene family expansions associated with immune function, energetic homeostasis, and wound healing. This reference genome will serve as a valuable resource for studies of evolution and environmental resilience in lizards.

## Introduction

Squamate reptiles are among the most species-rich group of tetrapods (Card et al. 2023). They constitute a key pillar in the evolution of amniotes and display an extraordinary diversity of phenotypic and genomic traits which makes them a fascinating group to study (Olmo 2023). Despite this, the development of genomic resources for non-avian reptiles has progressed slower than that of birds and mammals.

Reptile evolution is thought to have been shaped by adaptive responses to shifting climate (Simões et al. 2022). Lizards, in particular, have been useful in gaining an understanding of species responses to anthropogenic selection pressures (Winchell et al. 2023). The Australian water dragon (*Intellagama lesueurii*) is a large, semi-aquatic member of the Agamidae family of iguanian lizards that inhabits riparian landscapes along the east coast of Australia. It is the sister taxon to the core mesic Australian radiation (Amphibolurinae) (Hugall et al. 2008), the primitive body form typical also of *Hypsiluris* and *Physignathus*. Adult water dragons are sexually dimorphic with adult males becoming larger in size and developing a distinctive red coloring on the abdomen (Johnson et al. 2018). Australian water dragons are urban adaptors and have complex social systems; characteristics that have led them to become a model for investigations involving rapid evolution and social behavior. They have been shown to exhibit morphological and behavioral differentiation between urban landscapes and when compared with natural populations (Littleford-Colquhoun et al. 2017; Piza-Roca et al. 2020). Studies have revealed urban populations to be socially structured (Strickland et al. 2014) and plasticity in mating strategies has been observed among males that can differ greatly in levels of aggression (Baird et al. 2020) offering an elegant system to investigate genetic traits controlling dominance behaviors.

The water dragon also exhibits temperature-dependent sex determination (TSD) enabling detailed studies of the effects of environmental change on reproductive behavior where, for example, female water dragons have been shown to actively compensate for changes to the thermal environment by altering the depth of their nests (Jackson et al. 2019). This trait provides an excellent opportunity to study sex chromosome evolution and as heterogamety has not been established in this species, it enables comparative studies in conjunction with the only other published Agamid genome of *Pogona vitticeps* which exhibits a female heterogametic genetic sex-determining mechanism (Georges et al. 2015).

Further, water dragons may serve as a unique model for studies of the impacts of emerging infectious diseases on wildlife. With increasing concerns over novel pathogens infecting both captive and wild reptiles, recent outbreaks of fungal disease in urban water dragon populations present an ideal system for investigations of host responses to infection and for understanding population-level disease dynamics (Peterson et al. 2020; Tacey et al. 2023).

Unraveling the genomic architecture of this remarkable creature allows us not only to deepen our understanding of the evolution of the Agamidae but also to shed light on the genetic basis of its distinctive life history traits. Here we report a chromosome-level genome assembly, annotation, and comparative analysis with other high-quality squamate genomes to provide insight into the genomic innovations in this iconic Australian species.

## Results

### Chromosome-level genome assembly

**Fig. 1.**
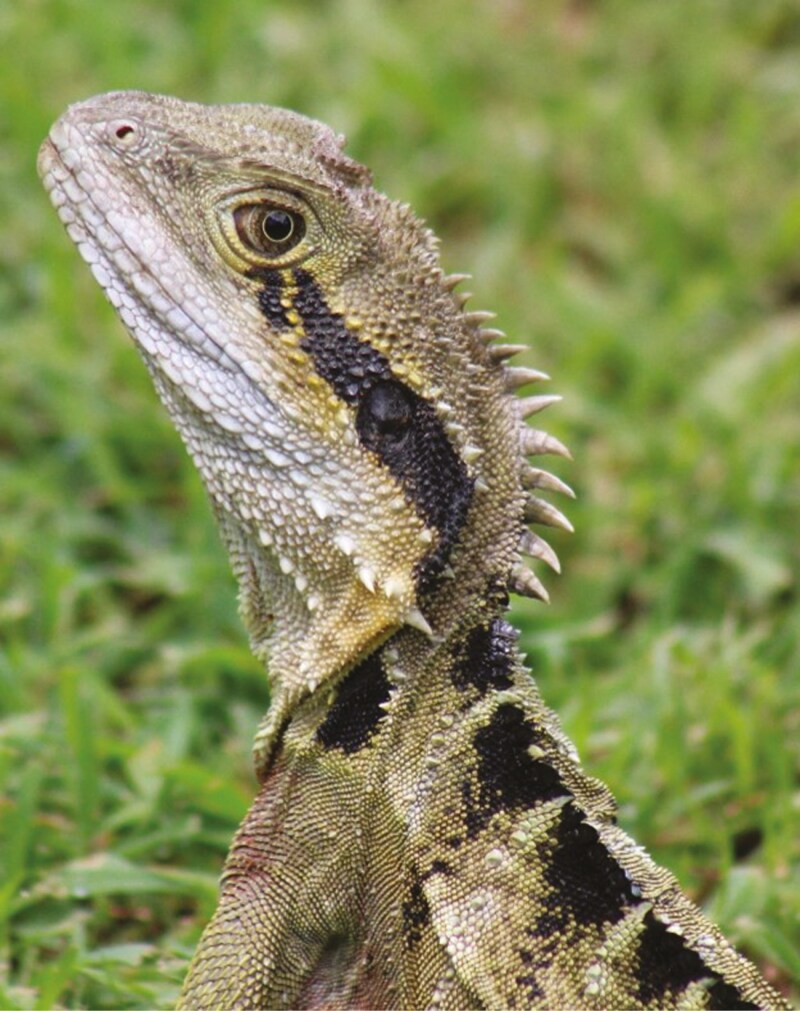
Picture of *November* the juvenile male eastern water dragon from Brisbane, Australia whose genome was sequenced in this study.

A total of 2.28 million reads were generated from a male eastern water dragon (Fig. 1) using two PacBio HiFi SMRT cells totaling 33.1 Gb of sequence data. An additional 107 million Illumina 150 bp paired-end reads were produced from an in situ Hi-C library using DNA from the sample animal.

The size of the eastern water dragon genome was estimated to be approximately 1.6 Gb based on the *k*-mer profile analysis of the Illumina resequencing data from both a male and female dragon using a *k*-mer size of 17 (Supplementary Figs. S1 and S2). The estimated genome size is slightly smaller than that of other closely related species such as the fence lizard (*Sceloporus undulatus*), however, the draft assembly size of 1.817 Gb is similar to the published genome assemblies of the closely related agamid *P. vitticeps* (1.716 Gb) and of *Anolis carolinensis* (1.799 Gb) (Table 1). Heterozygosity is estimated to be 0.322% for both male and female samples (Supplementary Figs. S1 and S2). The overall CG content is 41.91% which is similar to 41.81% for *P. vitticeps.* Repetitive sequence comprised 40.25% of the assembly (Supplementary Table S1).

**Table 1.** Genome assembly statistics for *Intellagama lesueurii* and comparative statistics for the closely related species *Pogona vitticeps* and *Anolis carolinensis*.

	IntLes1.0^a^	pvi1.1^b^	AnoCar2.0^c^
Total length of scaffolds	1,817.7 Mb	1,716.6 Mb	1,799.1 Mb
Percentage gap	0.009%	3.986%	5.435%
Number of contigs	875	98,807	41,987
Number of scaffolds	556	13,749	6,457
Contig N50	39.6 Mb	35.5 kb	79.9 kb
Scaffold N50	269.1 Mb	2.5Mb	150.6 Mb
Longest contig	93.4 Mb	295.8 kb	582.0 kb
Longest scaffold	353.5 Mb	14.7 Mb	263.9 Mb
GC content (%)	41.91%	41.81%	40.32%
Percentage genome in scaffolds >50 kb	99.67%	97.89%	96.43%
BUSCO (vertebrata_odb10)			
Complete and single copy	3,243 (96.7%)	3,190 (95.1%)	2,971 (87.4%)
Complete and duplicated	36 (1.1%)	23 (0.7%)	40 (1.2%)
Missing	47 (1.4%)	61 (1.8%)	216 (6.4%)

The table also includes BUSCO completeness statistics performed at the genome level for each of the three genome assemblies.

^a^This study. GenBank accession numbers:

^b^GCF_900067755.1,

^c^GCA_000090745.2.

**Fig. 2.**
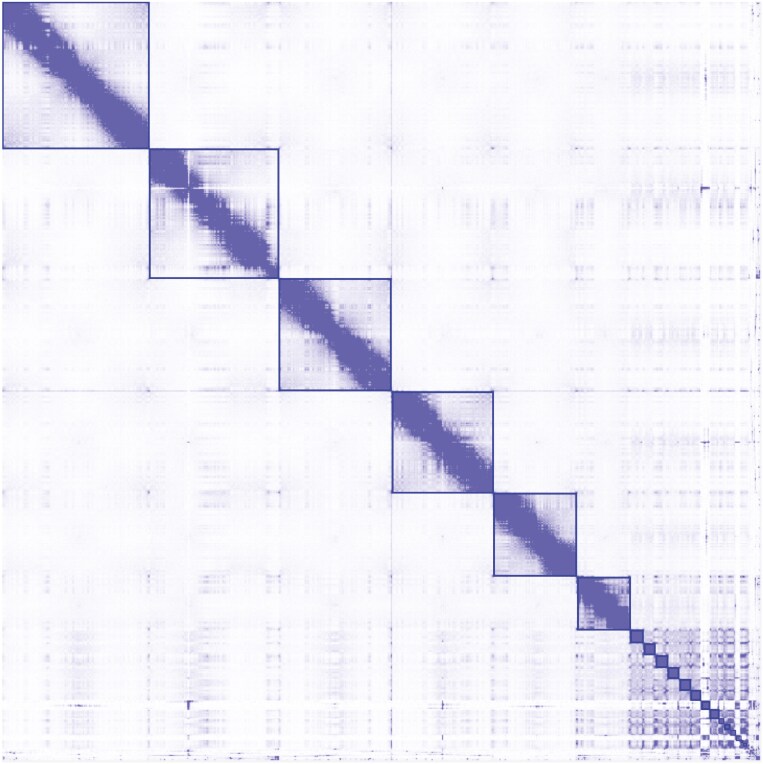
A Hi-C contact map highlighting the pairwise density between the six haploid *Intellagama lesueurii* macrochromosomes and showing a higher level of interchromosomal interaction between the scaffolds representing the 12 microchromosomes in the assembly. Together, these 18 scaffolds represent over 98.5% of the total 1.8 Gb assembly.

The eastern water dragon has an expected 2*n* chromosome number of 36 which includes 12 macro and 24 microchromosomes (Witten 1983). This karyotype represents an ancestral organization common in the Iguania and observed in Asian agamids (Young et al. 2013). As a basal member of the Australian agamid phylogeny (Hugall et al. 2008), the eastern water dragon also displays this ancestral genome structure. Our assembly includes six chromosome-length scaffolds (>127 Mb) representing the six haploid macrochromosomes and a further set of 16 scaffolds (between 31.6 and 1.1 Mb) that comprise the 12 haploid microchromosomes that together represent over 98.5% of the 1.8 Gb assembly (Fig. 2). There was a total of 556 scaffolds in the assembly resulting in an N50 of 269 Mb with the largest scaffold being 353 Mb in length (Table 1).

The completeness of the eastern water dragon genome assembly was assessed using the BUSCO vertebrate lineage dataset which resulted in the identification of 97.8% complete BUSCO genes with only 1.1% of these genes found in duplicate, suggesting a high degree of completeness and minimal redundancy for this draft. The overall alignment rate for resequenced Illumina clean reads were 99.22% and 99.26% for female and male samples, respectively, demonstrating the vast majority of genomic information was captured in the assembly.

Whole-genome comparison of the chromosome-length scaffolds from the *Intellagama* assembly showed a high degree of collinearity with the chromosome-length Hi-C assembly for *P. vitticeps*. In fact, 88.08% of the eastern water dragon genome aligned with between 25% and 50% similarity to the central bearded dragon genome (Supplementary Fig. S3). The *P. vitticeps* karyotype consists of six pairs of macrochromosomes but only 10 pairs of microchromosomes (Deakin et al. 2016), including the sex microchromosomes. There are three scaffolds that align to the largest microchromosome in *P. vitticeps* and given the number of large-scale scaffolds exceeds the observed number of chromosomes, we expect a small degree of fragmentation to remain in our assembly.

*Pogona vitticeps* genomics scaffolds scf000160, scf000179, scf000280, and scf000531 that were anchored to the Z chromosome in a previous study (Deakin et al. 2016) were aligned to *Pogona* Hi-C_scaffold_16 with 96.8% pairwise identity across an 8.33 Mb region of the 11.49 Mb total length indicating this Hi-C scaffold likely represents the male sex chromosome. This *Pogona* Hi-C_scaffold_16 shows high levels of collinearity with *Intellagama* HiC_scaffold_17 (Supplementary Fig. S4) indicating the genomic structure of this microchromosome has remained conserved despite persisting in a species with temperature-dependant sex determination.

**Fig. 3.**
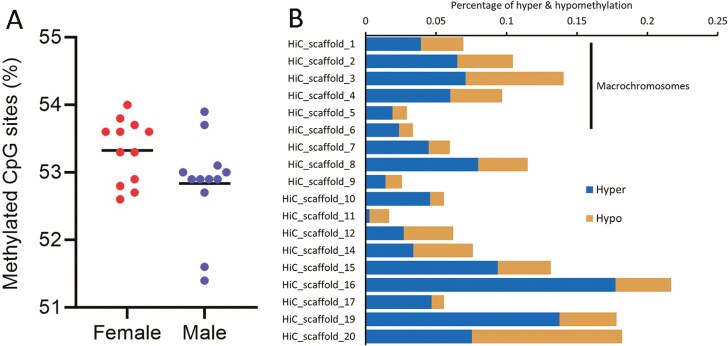
Epigenomic sex differences in the *Intellagama lesueurii* genome. (A) Comparison of DNA methylation pattern in whole blood between female and male water dragons (female mean = 53.33; male mean = 52.83; *P*-value 0.0590). The horizontal line indicates the mean for each dataset. (B) The percentage of differentially methylated sites (DMS) identified between male and female water dragons. The majority (60.3%) were observed as hypermethylated in female samples. The microchromosomes generally exhibited a higher degree of hypermethylation than the macrochromosomes.

Variation in DNA methylation was measured across 12 female and 12 male dragons to investigate the presence of sex differences that may influence gene expression levels. We find that female dragons have a slightly higher albeit not significantly different average DNA methylation in whole blood compared with males (Fig. 3A). However, a total of 1,153 CpG sites were determined to be significantly differentially methylated between females and males across a total of 1.33 million CpG sites common to all samples. Of these differentially methylated sites (DMS), the majority (60.3%) were hypermethylated in female samples with the microchromosomes generally exhibiting a higher degree of hypermethylation than the macrochromosomes (Fig. 3B).

### Genome annotation

To provide support for gene model predictions, we generated RNA-Seq libraries from nine different tissues that were sequenced to produce over 680 million paired-end short reads. These short reads were mapped to the genome assembly with an overall alignment rate of 94.12%. We modeled a final set of 23,675 protein-coding genes by combining homologous protein information, de novo prediction, and transcriptomic read alignment, with 20,284 of these genes assigned homologs in other species of squamates and 16,885 containing at least one Pfam domain. We identified 93.5% complete core vertebrate genes from the BUSCO database among the annotated gene set which is comparable with other high-quality squamate genomes published to date (Supplementary Fig. S5). The number of gene models and the statistics of their genomic features are similar to those described for *P. vitticeps* (Table 2).

**Table 2.** Statistics of the gene model predictions for the *Intellagama lesueurii* genome described in this study in comparison with the NCBI RefSeq annotations of *Pogona vitticeps*.

Description	IntLes1.0	pvi1.1.0^a^
Number of genes	23,675	21,994
Total CDS length (bp)	35,255,157	74,249,862
Shortest gene (bp)	156	71
Longest gene (bp)	3,729,048	1,514,754
Longest CDS (bp)	104,847	106,293
Mean gene length (bp)	36,071	38,825
Mean exon length (bp)	414	262
Mean intron length (bp)	3,977	4,712
Mean CDS length (bp)	1,489	1,918
% of genome covered by genes	47	50
Number containing Pfam domains	16,885	16,667

^a^GenBank accession GCF_900067755.1.

### Comparative analysis

**Fig. 4.**
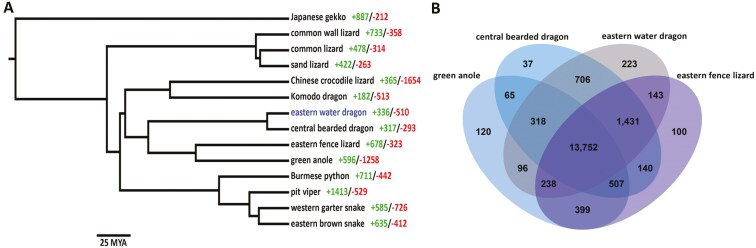
Comparative analysis of predicted squamate protein-coding sequences. (A) Phylogenetic analysis showing the taxonomic position of the eastern water dragon inferred using single copy orthologs found across all included species together with the ortholog gain (+) and loss (-) identified for each of the squamate species included in this study from the publicly available annotated squamate gene sets. The scale bar represents 25 million years ago. (B) The overlap of shared orthologous gene groups between the eastern water dragon and the three most closely related squamate reptile species.

Using the longest protein-coding sequences from the full set of genes annotated from 14 high-quality squamate genomes (Supplementary Table S2), 273,224 genes (97.3% of the total) were assigned to 19,403 orthogroups. Fifty percent of all genes were in orthogroups with 14 or more genes. There were 11,089 orthogroups that contained at least one gene from all species and 6,614 of these consisted entirely of single-copy genes. A phylogenetic tree was constructed for the 14 species of Squamata using the protein sequences from the 6,614 single-copy orthologues that were present in all species. The eastern water dragon and the central bearded dragon were determined to have diverged from a common ancestor approximately 16 million years ago (Fig. 4A). When comparing the *Intellagama* genome with the three most closely related species in this study, *P. vitticeps*, *A. carolinensis*, and *S. undulatus*, we identified 13,752 orthogroups common to all four of these species. However, there are 223 orthogroups found to be unique to the *Intellagama* genome (Fig. 4B). Within this set of unique orthogroups, there were seven Gene Ontology (GO) terms found to be enriched notably, immune response, response to pheromone, and detection of mechanical stimulus (Supplementary Table S3).

Changes in gene family sizes in *Intellagama* were analyzed using both protein orthology and protein domain occurrence. Branch-specific expansion of the orthologous gene groups as determined by CAFE analysis identified 336 expanded and 510 contracted gene families (Fig. 4A). GO enrichment analysis of the 336 orthologous gene groups gained in *I. lesueurii* resulted in 12 enriched GO terms that are associated with immune function, cell signaling, energetic homeostasis, and reproduction (Table 3). Further, we identified the expansion of 20 protein family domains (Supplementary Fig. S6) that show some overlap with the expanded orthologous gene groups from the CAFE analysis. Notably, those associated with immune function and cell signaling. Through their role in inflammation, several expanded orthologous gene groups associated with immune activation could subsequently be linked to wound healing, with expression of these gene groups concentrated in the skin and spleen (Supplementary Fig. S7).

**Table 3.** The enriched gene ontology (GO) terms identified from the 336 orthologous gene groups found to be expanded in the *Intellagama lesueurii* genome are associated with immune function, cell signaling, energetic homeostasis, and reproduction.

GO ID	GO name	FDR
GO:0019882	Antigen processing and presentation	3.4E−24
GO:0007156	Homophilic cell adhesion via plasma membrane adhesion molecules	1.7E−20
GO:0010185	Regulation of cellular defense response	5.30E−03
GO:0001868	Regulation of complement activation, lectin pathway	5.30E−03
GO:0007194	Negative regulation of adenylate cyclase activity	7.80E−03
GO:0007093	Mitotic cell cycle checkpoint signaling	1.10E−02
GO:0006707	Cholesterol catabolic process	2.70E−02
GO:0140029	Exocytic process	4.50E−02
GO:0007276	Gamete generation	5.00E−02
GO:0046314	Phosphocreatine biosynthetic process	5.00E−02
GO:0060122	Inner ear receptor cell stereocilium organization	5.00E−02

**Fig. 5.**
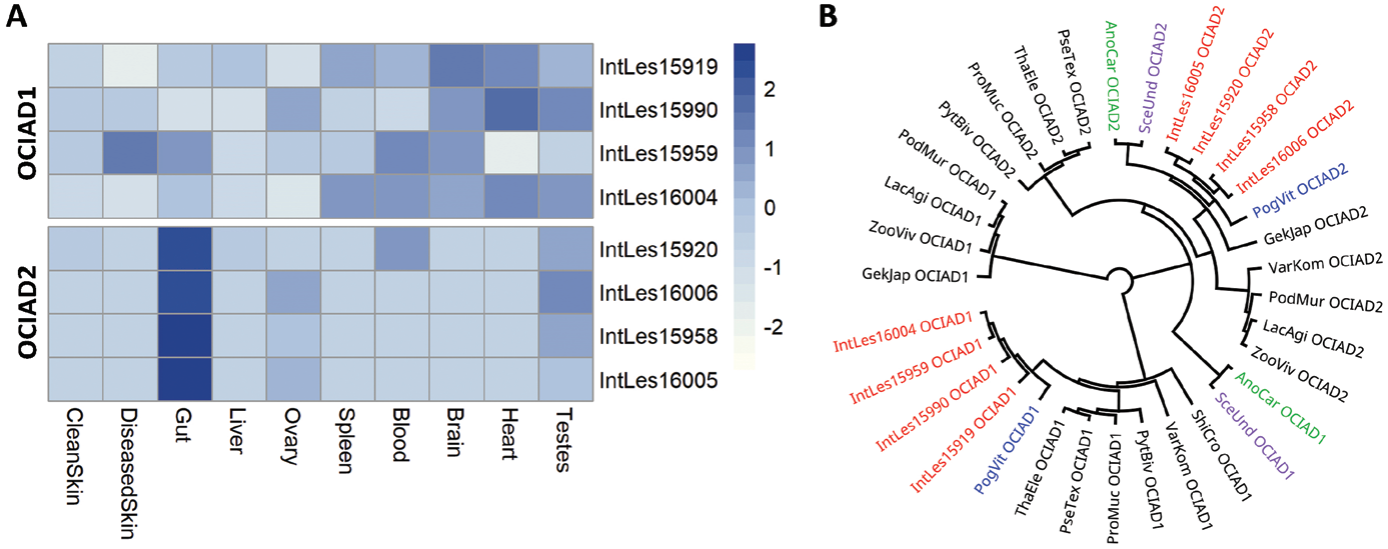
Analysis of the OCIAD gene families in *Intellagama lesueurii*. (A) The tissue-specific gene expression profile of both OCIAD gene families in *I. lesueurii* highlights the localization of OCIAD2 in the gut and the more general tissue distribution for OCIAD1. The scale bar represents the row *Z-*score. (B) A phylogenetic analysis of protein-coding sequences of the expanded OCIAD gene families in *I. lesueurii* (IntLes) together with the OCIAD genes from the other squamate reptiles included in this study. Both OCAID gene families cluster together with the closely related *Pogona vitticeps* (PogVit), *Anolis carolinensis* (AnoCar), and *Sceloporus undulatus* (SceUnd).

Common to both the gained orthologous gene groups and the expanded protein family domains were the Ovarian Cancer Immunoreactive Antigen Domain Containing (OCIAD) genes. Each of the other 13 species included in our genomic comparison contained just one copy of the genes OCIAD1 and OCIAD2, whereas the *Intellagama* genome contained four copies of each gene, all located on macrochromosome Hi-C_scaffold_5. The expression of the OCIAD1 gene family differed between tissues with higher levels seen in the brain, heart, blood, and testes (Fig. 5A), while the expression of the OCIAD2 gene family was mainly concentrated in the gut. The protein-coding sequences from these genes clustered closely with each other and are most similar to the OCIAD genes from *Pogona* (Fig. 5B).

## Discussion

Genomic resources for non-avian reptiles are underrepresented when compared with mammals and birds (Pinto et al. 2023). Here we report a chromosome-length genome assembly of *I. lesueurii* and accompanying annotations together with extensive transcriptomic and epigenomic datasets. Our evaluation of this draft assembly suggests a high degree of completeness and minimal redundancy supporting its usefulness as one of very few publicly available reference genomes for the Agamidae family of lizards. The resulting gene annotations have enabled the first genome-level comparison between agamid lizards with the previously reported *P. vitticeps* enabling an estimation of divergence time to 16 MYA using a comprehensive set of over 6,000 protein-coding sequences.

### Sex differences

The evolution of sex determination in Squamata is complex and has involved multiple transitions between different mechanisms (Pokorná and Kratochvíl 2009). The close phylogenetic relation of *I. lesueurii to P. vitticeps* offers an interesting comparison between species with differing sex-determination systems. The Australian central bearded dragon has a female heterogametic ZZ/ZW system of genetic sex determination. However, mating experiments of naturally occurring sex-reversed males with normal males resulted in an all-ZZ generation of which any offspring was determined in a temperature-dependent manner (Holleley et al. 2015). As a result, the W chromosome was seen to be eliminated from a lineage within a single generation. Alignment of resequencing data from both male and female water dragons showed similar profiles across each of the larger scaffolds in the genome assembly providing no direct evidence of sex chromosomes in this species. However, the high level of collinearity observed between the likely Z chromosome scaffold in the recently constructed *P. vitticeps* genome assembly and *Intellagama* Hi-C_scaffold_17 suggests that the establishment of this genomic region likely preceded the transition of either species to a different mode of sex-determination from the common ancestor. Whether a W chromosome had been lost from *I. lesueurii* over the course of time in a similar manner to what has been demonstrated for *P. vitticeps* remains to be determined.

DNA methylation is an epigenetic mechanism that can play an important role in regulating gene expression in sex-determination pathways in vertebrates with environmental sex-determination systems (Shao et al. 2014). Higher temperatures have been shown to increase the level of DNA methylation in female fish, inducing masculinization (Navarro-Martín et al. 2011). We used non-lethal whole blood sampling to explore whether there may be sex-biased differences in DNA methylation in *I. lesueurii* as observed in skin samples from the green sea turtle (*Chelonia mydas*) well known for exhibiting TSD (Mayne et al. 2023). We observed a somewhat higher level of overall DNA methylation in female dragons. Interestingly, we also found an increase in the hypermethylation of DMS in females that was most pronounced in the microchromosomes, with the highest ratio of hyper to hypomethylation occurring on Scaffold 17. While further studies are required to investigate methylation patterns during gonadal development, these differences may enable the development of a low-cost molecular assay for sex individuals at earlier life stages where dimorphism is not yet apparent.

### Genomic innovations

Central to the gene group expansions identified in the *I. lesueurii* genome are genes involved in the immune response, in particular antigen recognition and presentation. These genes facilitate the ability of squamates to respond to the wide variety of pathogens encountered throughout the diverse range of environments they inhabit. Consistent with reports from other squamate species, there were notable expansions of genes involved in immune system activation in the *I. lesueurii* genome. The immunoglobulin (Ig), major histocompatibility complex (MHC), and C-type lectin (CLR) gene families were found to be larger in size than the other species included in this study. However, the size of the Ig and MHC expansions resembled those reported for *A. carolinensis* (Das et al. 2008; Card et al. 2022). As these reports were based on sequences annotated via manual curation, the full complement of genes from these families may not have been captured in the gene model annotations accompanying the publicly available genome drafts. This is likely due to the difficulties of automated gene predictors to adequality resolve these regions containing high gene density and duplications.

The gene groups of serpin B3, serpin B4, angiogenin, cathepsin L1, and interleukins IL-10 and IL-20 were each found to be expanded in the *Intellagama* genome. The interplay between these gene groups lies in their relationship with the inflammatory response, which serves as a critical phase in the process of wound healing in reptiles, helping to clear debris, fight off infections, and initiate tissue repair. Serpins B3 and B4 have been shown to inhibit cathepsins and elevation of these have been detected during inflammation suggesting they may be upregulated to help suppress the inflammatory response (Kelly-Robinson et al. 2021). They can also promote the production of interleukins to elicit a pro-inflammatory response (Sheshadri et al. 2014) and as such, serpins may act as modulators potentially influencing the magnitude and duration of inflammatory reactions. Angiogenin is induced during inflammation (Tello-Montoliu et al. 2006) and promotes the formation of blood vessels which is an important component of wound repair (Lyons et al. 2017). Cathepsins also have a role in angiogenesis through the regulation of endothelial cell signaling and mediating cytokine maturation (Liu et al. 2018). The IL-10 family includes IL-20 which are cytokines that can inhibit inflammation and also promote wound healing (Rutz et al. 2014; Short et al. 2022). Both can be expressed in keratinocytes (Blumberg et al. 2001) which are major components of skin wound repair after injury. Interestingly, several keratin gene families were also expanded in the *Intellagama* genome. Keratins are expressed by keratinocytes and play a role in providing structural support and integrity to migrating and proliferating cells during wound healing (Ramms et al. 2013; Zhou et al. 2023). Taken together, we find many of the expanded gene families identified in *Intellagama* can be linked to wound healing either directly or via their involvement in inflammatory processes. This is further supported by the concentrated expression of these gene groups in the skin and spleen (Supplementary Fig. S7). Territorial male water dragons are highly combative and regularly engage in fierce contests with competing males (Baird et al. 2014), often inflicting severe wounds that can become fatally infected. Likewise, females may also sustain injuries during aggressive courtship or mating interactions with males (Baxter-Gilbert and Whiting 2019). These orthologous gene group expansions observed in this study may be characteristic of an increased capacity for wound healing that may have developed in response to such life history traits. Similar adaptations involving keratin proteins and genes involved in angiogenesis have been observed in the genome of sharks also known for their remarkable wound-healing abilities (Marra et al. 2019).

The OCIAD family genes were each found in four copies in the *Intellagama* genome compared with just one copy found in all other squamate species included in this study. OCIAD1 and OCIAD2 usually occur on opposite DNA strands oriented tail-to-tail in vertebrates (Praveen et al. 2020) and this same pairing is observed for each of the four pairs indicated to be the sequential gene numbering seen in Fig. 5. These genes encode membrane-bound proteins that localize to the mitochondria where OCIAD1 has been shown to regulate mitochondrial complex I activity (Shetty et al. 2018), and where both OCIAD1 and OCIAD2 were found to be required for assembly of mitochondrial complex III (Le Vasseur et al. 2021; Chojnacka et al. 2022), which are both integral components of the electron transport chain (ETS). OCIAD1 has been shown to regulate energy metabolism in human pluripotent stem cells (Shetty et al. 2018) and suppression of OCIAD2 led to a reduction in mitochondria and the downregulation of cellular growth in cancer cell lines (Hong et al. 2021). Animal mitochondria are sensitive to thermal stress (Sokolova 2018) and the capacity of this organelle to generate energy is highly influenced by temperature (Lemieux and Blier 2022). Favoring riparian habitats, water dragons often seek refuge in water once disturbed. Upon entering the water, a large dragon could rapidly cool to 10 to 12 °C lower than their preferred body temperature within around 10 min and they may stay there for up to 2 h (Courtice 1981). These expanded OCIAD genes could potentially offer an adaptive advantage to thermal stress by supporting a higher level of energy production at lower temperatures. Genes enabling a degree of metabolic flexibility would be advantageous as was proposed upon the discovery of gene variants from the two co-occurring mitochondrial genomes identified in the cold-tolerant tuatara (Macey et al. 2021).

Finally, these genomic resources of this iconic Australian reptile will further support research involving this emerging model organism for studies involving urban adaption, social behavior, and accelerated evolution as increasing global temperatures and expanding urban development continue to influence our wild populations.

## Methods

### Sampling and genome sequencing

A juvenile (sub-adult) male eastern water dragon (*I. lesueurii lesueurii)* named *November* collected from the Roma Street Parklands in Brisbane, Australia was euthanized on 29 March 2018 due to poor body condition. Blood samples were taken and stored in heparinized tubes frozen at −80 °C prior to high-molecular-weight DNA extraction using a spooling method in conjunction with the Puregene DNA Purification Kit (Qiagen). Fragmentation was performed to 20 kb using a Megaruptor 2 (Diagenode) and HiFi SMRTbell libraries were prepared using SMRTbell Express Template Prep Kit 2.0 before sequencing on a PacBio Sequel II system at the Australian Genome Research Facility. Blood from an additional adult male and female eastern water dragon from the same population was sampled and extracted using the Blood and Tissue Kit (Qiagen) for resequencing using the DNA PCR-free library prep and sequenced on the NovaSeq 500 to produce 150 bp paired-end reads at the Australian Genome Research Facility.

To produce a chromosome-length genome assembly, a frozen liver sample was used to construct in situ a Hi-C library as described in Rao et al. (2014). A total of 138,592,561 paired-end (150 bp) Hi-C reads were generated using NovaSeq 6000 (Illumina). The Hi-C library and reads were generated by the DNA Zoo Consortium.

Total RNA was extracted using the RNeasy Plus Mini kit (Qiagen) following the manufacturer’s instructions. Purified RNA was screened using a 2100 Bioanalyzer (Agilent) and extractions resulting in RNA Integrity Numbers (RINs) greater than 8.0 were selected for library preparation using the TruSeq Stranded mRNA library kit. Samples from the brain, testes, and heart, of the dragon *November* were sequenced on the Illumina NextSeq 500 to produce 75 bp paired-end reads at the Ramaciotti Centre for Genomics, Australia. Samples of liver, gut, ovary, spleen, blood, and skin taken from additional euthanized dragons from the Roma Street Parklands were sequenced at the Australian Genome Research Facility using the Illumina NovaSeq platform to generate 150 bp paired-end reads.

All animal handling and procedures were conducted in accordance with the University of the Sunshine Coast Animal Ethics approval (ANS1858 and ANA20161).

### Assembly workflow

Primary assembly of the PacBio HiFi CCS reads was performed using Hifiasm v0.14-r312 (Cheng et al. 2021) with the default parameters except for -k 53 -s 0.7 -r 6. Hi-C data was aligned to the draft reference using Juicer v1.6 (Durand et al. 2016). Hi-C guided scaffolding was performed using 3D-DNA v201008 (Dudchenko et al. 2017), and reviewed using Juicebox Assembly Tools (Dudchenko et al. 2018). The Hi-C assembly was further processed to remove redundant scaffolds with a similarity of 98% or greater before using TGS-GapCloser v1.0.1 (Xu et al. 2020) with the high-quality HiFi CCS reads to fill gaps from joins made from scaffolding. BUSCO v5.1.3 software (Manni et al. 2021) and the vertebrata_odb10 database were used to assess the level of completeness. Genome size estimation was performed using data from the short-read genome survey and *k*-mer counting with Jellyfish v2.3.0 (Marçais and Kingsford 2011) using GenomeScope 2.0 (Ranallo-Benavidez et al. 2020).

### Genome annotation

Expression levels were measured by aligning quality-processed RNA-Seq reads to the genome assembly using HiSat2 v2.1.0 (Kim et al. 2015). Aligned reads were sorted with SAMtools v1.5 (Li et al. 2009) and counts were reported as transcripts per one million mapped reads (TPM) using StringTie2 v2.2.0 (Pertea et al. 2016). TPM values visualized in heatmaps were transformed to log_2_ (TPM + 1) and normalized across tissues using the scale function in R.

RepeatMasker (v4.1.2-p1) (Smit et al. 2015) was used to soft mask the assembly prior to gene model prediction using a custom repeat database generated using RepeatModeler (v2.0.1) (Flynn et al. 2020) together with the Repbase library. Protein alignments from *Pogona*, *Anolis*, *Lacerta*, Chicken, and Human protein sets were obtained using an implementation of Fgenesh++ (Softberry, Inc.) (Solovyev et al. 2006) and the RNA-Seq alignment information from StringTie2 was incorporated into the MAKER v3.01.03 (Holt and Yandell 2011) gene annotation pipeline. Gene model predictions were retained if they were greater than 33 amino acids in length and were supported by at least one form of supporting evidence of either 1) a BLASTp match (*E*-value < 10^−10^) to the non-redundant protein GenBank database, 2) coverage by RNA-Seq data alignment, or 3) containing a hit to the Pfam database. BUSCO software was used in protein mode to assess the completeness of the predicted protein sequences from the gene models together with the protein sets from the other species of Squamata listed in Supplementary Table S2.

### Comparative genomics

A chromosome-length draft assembly for *P. vitticeps* was obtained from DNA Zoo (https://www.dnazoo.org/assemblies/Pogona_vitticeps) that was based on a previously sequenced individual (Georges et al. 2015). Whole-genome alignments were performed between *Intellagama* and *Pogona* Hi-C assemblies using Minimap2 v2.24 (Li 2018) and the output was visualized using D-GENIES (Cabanettes and Klopp 2018). Individual scaffolds were aligned using the Mauve v1.1.3 (Darling et al. 2010) plugin in Geneious Prime v 2023.2.1 (Biomatters Ltd.).

Protein sequences from *I. lesueurii, P. vitticeps*, *A. carolinensis*, and *S. undulatus* were compared for orthology using all-against-all alignments (*E*-value of 10^−5^) and clustered using OrthoVenn2 (Xu et al. 2019) with an inflation value of 1.5. The unique set of 223 *Intellagama*-specific gene clusters identified from this comparison were assigned GO terms using the Uniprot database and GO term enrichment was computed using a hypergeometric distribution in OrthoVenn2.

Phylogenetic inference from orthologous genes was undertaken by comparing the *I. lesueurii* gene set with the protein-coding sequences from 13 other species of Squamata (Supplementary Table S2) using OrthoFinder v2.4.0 (Emms and Kelly 2019). Single-copy orthologous gene groups identified in this analysis were used to build an ultrametric maximum likelihood-rooted species-tree from multiple sequence alignments using the inbuilt MAFFT (Katoh and Standley 2013) alignment option and IQ-TREE 2 v2.2.0 (Minh et al. 2020). The tree was rooted using the Japanese gecko (*Gekko japonicus*) and calibrated with the TimeTree 5 divergence time estimates (Kumar et al. 2022).

In order to identify expansions of gene families within the *Intellagma* genome, we searched the protein-coding sequences for Pfam domains to assign gene function. We used HMMER v3.1 (Potter et al. 2018) (hmmscan) to search the Pfam A database (release 34.0) (Finn et al. 2014) for 19,179 different domains. Counts of each domain were collated for each species and domains that occurred multiple times in a protein sequence were counted only once. We used CAFE v4.2.1 (Han et al. 2013) using the default *P*-value thresholds to examine the expansion and contraction of the orthologous gene groups identified with OrthoFinder. Protein-coding sequences of the OCIAD genes present in the species of Squamata used in this study were aligned using MAFFT and visualized using the FastTree v2.1.12 (Price et al. 2010) plugin in Geneious Prime.

### Methylation analysis

DNA was purified from blood samples stored frozen at −80 °C in heparinized tubes using the Blood and Tissue Kit (Qiagen). Reduced Representation Bisulfite Sequencing (RRBS) with the restriction enzyme *MspI* was undertaken using the NuGEN Ovation RRBS Methyl-Seq system (Tecan Group Ltd.) and sequenced on the Illumina NovaSeq. General adaptor and quality trimming were performed with TrimGalore v0.6.7 before further diversity adaptor trimming and filtering using scripts from the NuMetRRBS GitHub repository (https://github.com/nugentechnologies/NuMetRRBS). Clean read data were aligned to the *Intellagama* genome using Bismark v0.22.3 (Krueger and Andrews 2011) and PCR duplicates were identified and removed using the script nudup.py. Differential methylation was analyzed using the methylKit v 1.20.0 (Akalin et al. 2012) pipeline in R. CpG sites were considered to be significantly differentially methylated between sexes if they were calculated to have a *q*-value of 0.01 and a percent methylation difference larger than 25%. Overall methylation was calculated for each sample by adding the percentage methylation at each site and dividing by the total number of sites. A comparison of global methylation levels between female and male dragons was performed using an unpaired *t-*test.

## Supplementary material

Supplementary material is available at *Journal of Heredity* online.

*Conflict of interest statement*. None declared.

esae054_suppl_Supplementary_Figures_S1-S7_Tables_S1-S3

## Data Availability

Pacbio HiFi sequencing data is available via the AusARG Bioplatforms data portal https://data.bioplatforms.com/organization/about/ausarg. The whole-genome assembly described in this paper has been deposited at DDBJ/ENA/GenBank under the accession JBAJHW000000000. All raw sequencing data is deposited under the NCBI BioProject accession number PRJNA1048105. Interactive Hi-C contact maps are available at www.dnazoo.org. All other supporting data can be accessed at https://doi.org/10.6084/m9.figshare.24784176.v1.
