# Supplementary material for: The genome of the Australian water dragon (Intellagama lesueurii), an agamid model for urban adaptation
Source: J Hered. 2024 Oct 4;116(5):571–81. doi: 10.1093/jhered/esae054 (PMC12400805; doi:10.1093/jhered/esae054)
Supplement: esae054_suppl_Supplementary_Figures_S1-S7_Tables_S1-S3 [file esae054_suppl_supplementary_figures_s1-s7_tables_s1-s3.pdf]

The genome of the Australian water dragon (*Intellagama lesueurii*), an agamid model for urban adaptation.

Daniel Powell[1,2*], Nicola Jackson[1], Parwinder Kaur[3], Olga Dudchenko[4,5], Erez Lieberman Aiden[4,5,6], Arthur Georges[7] and Céline, H. Frère[1]

[1]School of the Environment, University of Queensland, St Lucia, QLD, Australia

[2]Centre for Bioinnovation, University of the Sunshine Coast, Sippy Downs, QLD, Australia

[3]UWA School of Agriculture and Environment, The University of Western Australia, Perth, WA, Australia

[4]The Center for Genome Architecture, Department of Molecular and Human Genetics, Baylor College of Medicine, Houston, TX, USA

[5]The Center for Theoretical Biological Physics, Rice University, Houston, TX, USA

[6]Broad Institute of MIT and Harvard, Cambridge, MA, USA

[7]Institute for Applied Ecology, University of Canberra ACT 2601 Australia

Supplementary Material

Supplementary Figures

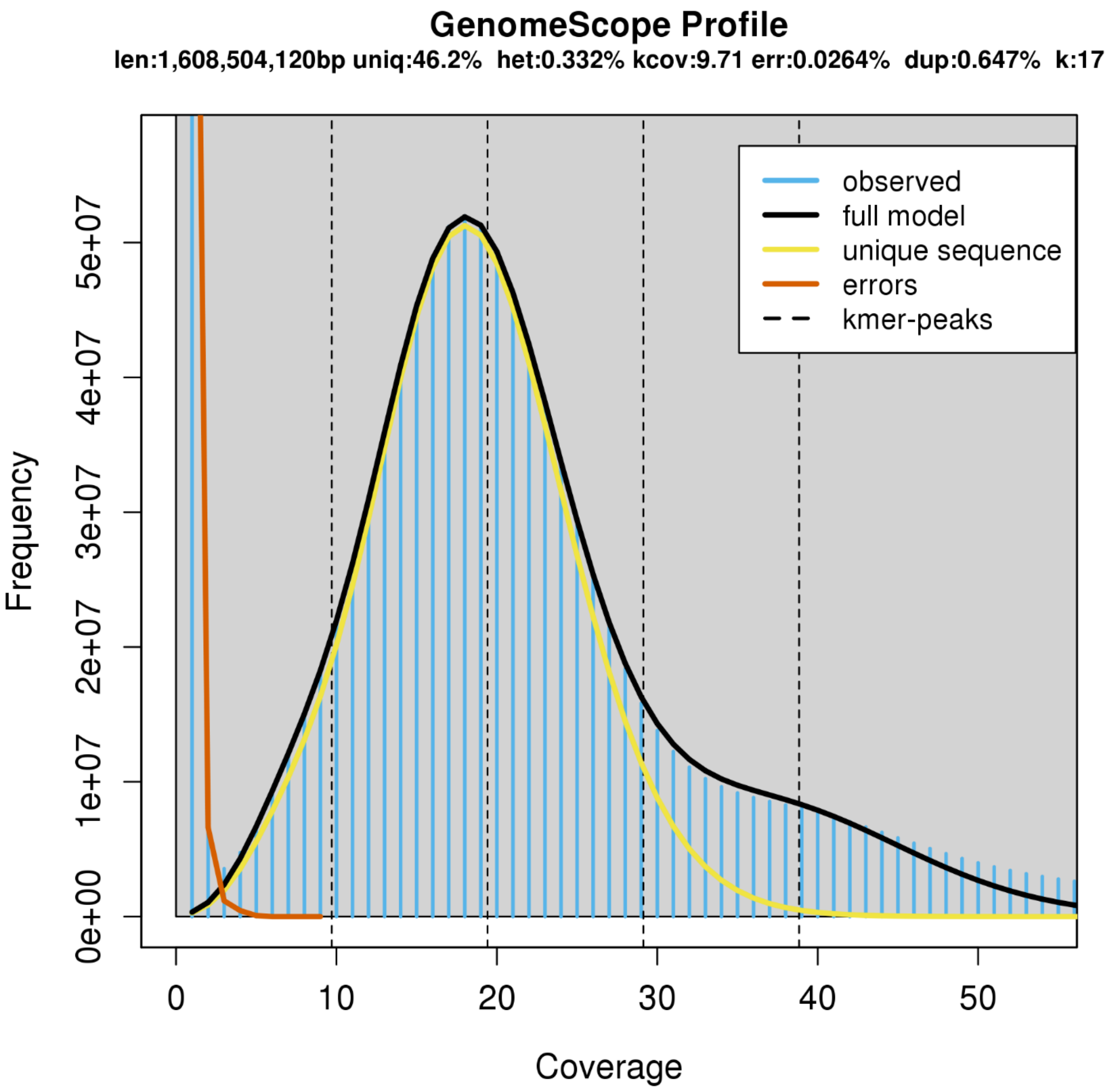

Supplementary Figure S1. Genome size estimation based on *k*-mer profile of a male dragon performed using Illumina short-read data.

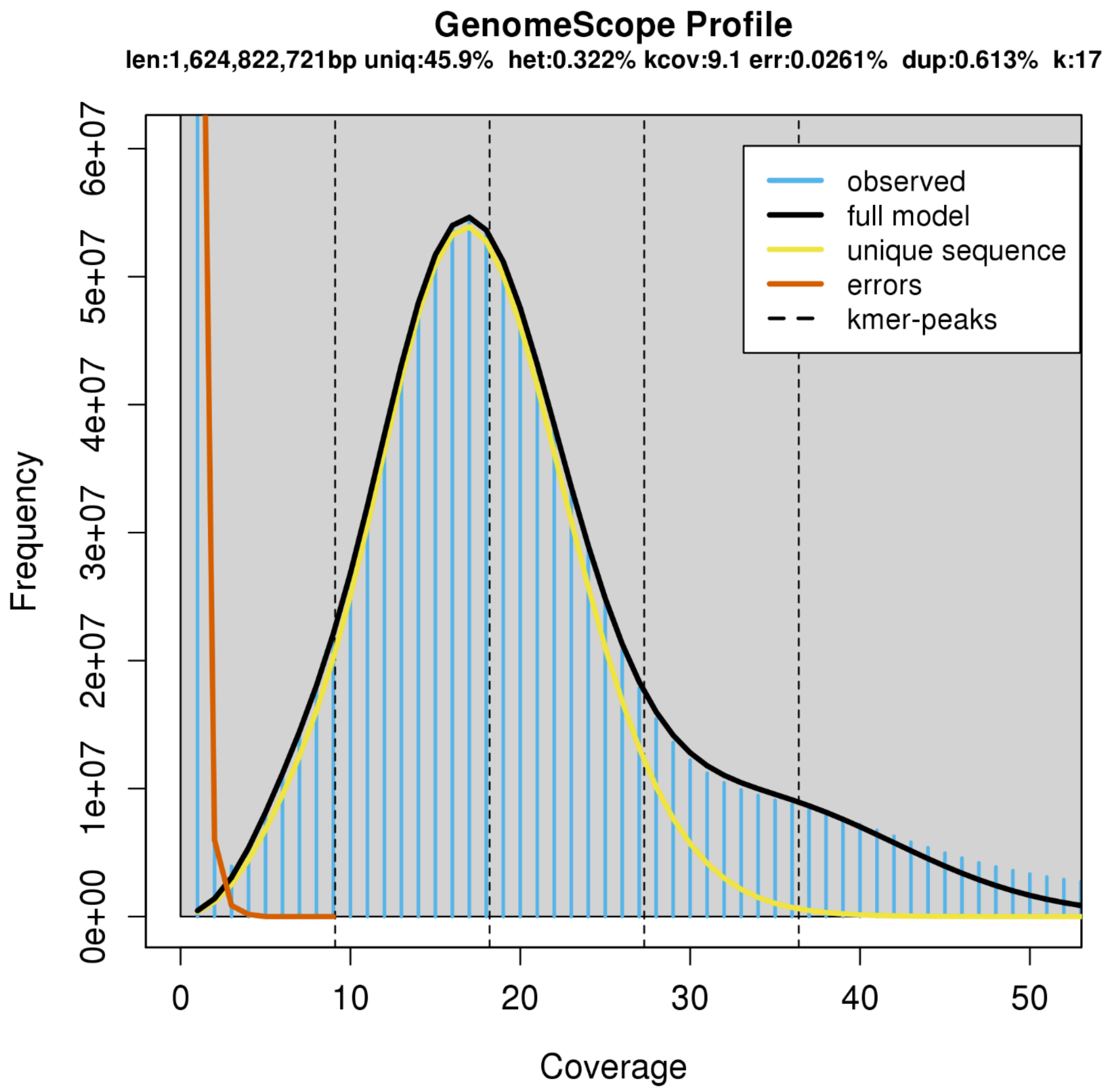

Supplementary Figure S2. Genome size estimation based on *k*-mer profile of a female dragon performed using Illumina short-read data.

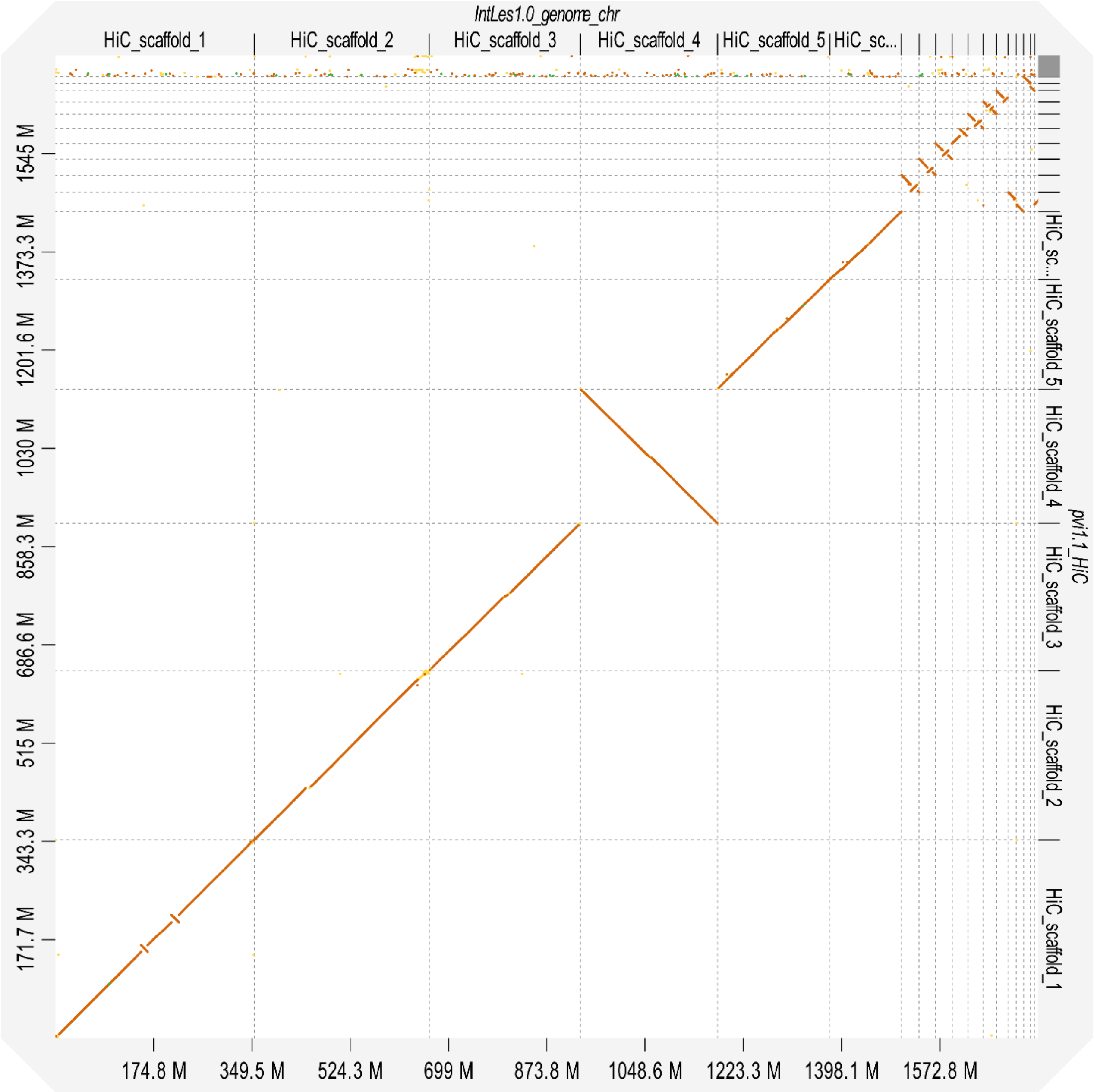

Supplementary Figure S3. Dotplot alignment of the chromosome-scale scaffolds of *I. lesueurii* when compared with *Pogona vitticeps*. Scaffold 7 representing the largest of the microchromosomes in *P. vitticeps* is split across 3 scaffolds in *I. lesueurii.*

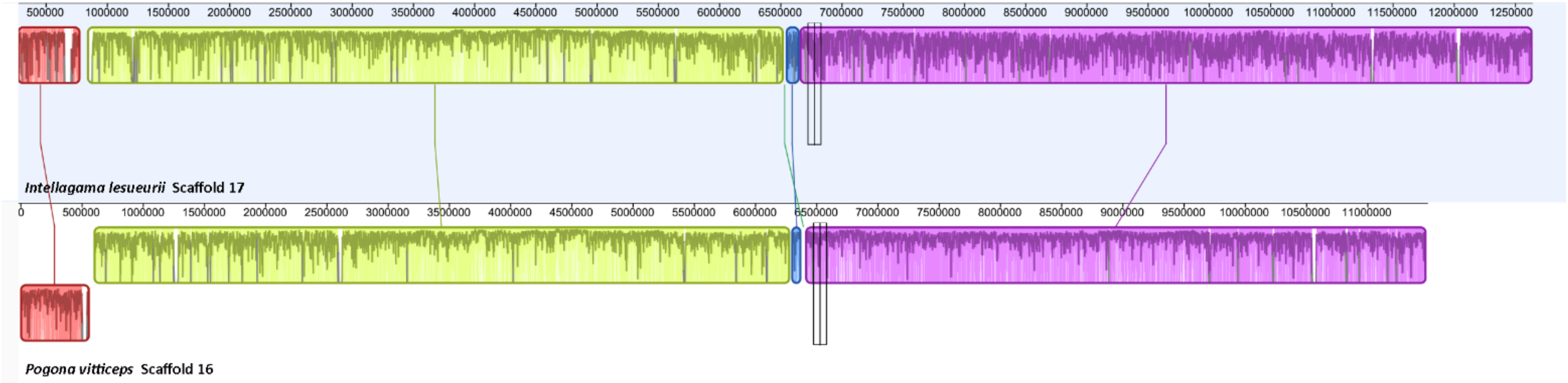

Supplementary Figure S4. The level of **c**ollinearity observed between the likely Z chromosome Hi-C scaffold in *P. vitticeps* (Scaffold 16) and the *I. lesueurii* Scaffold 17.

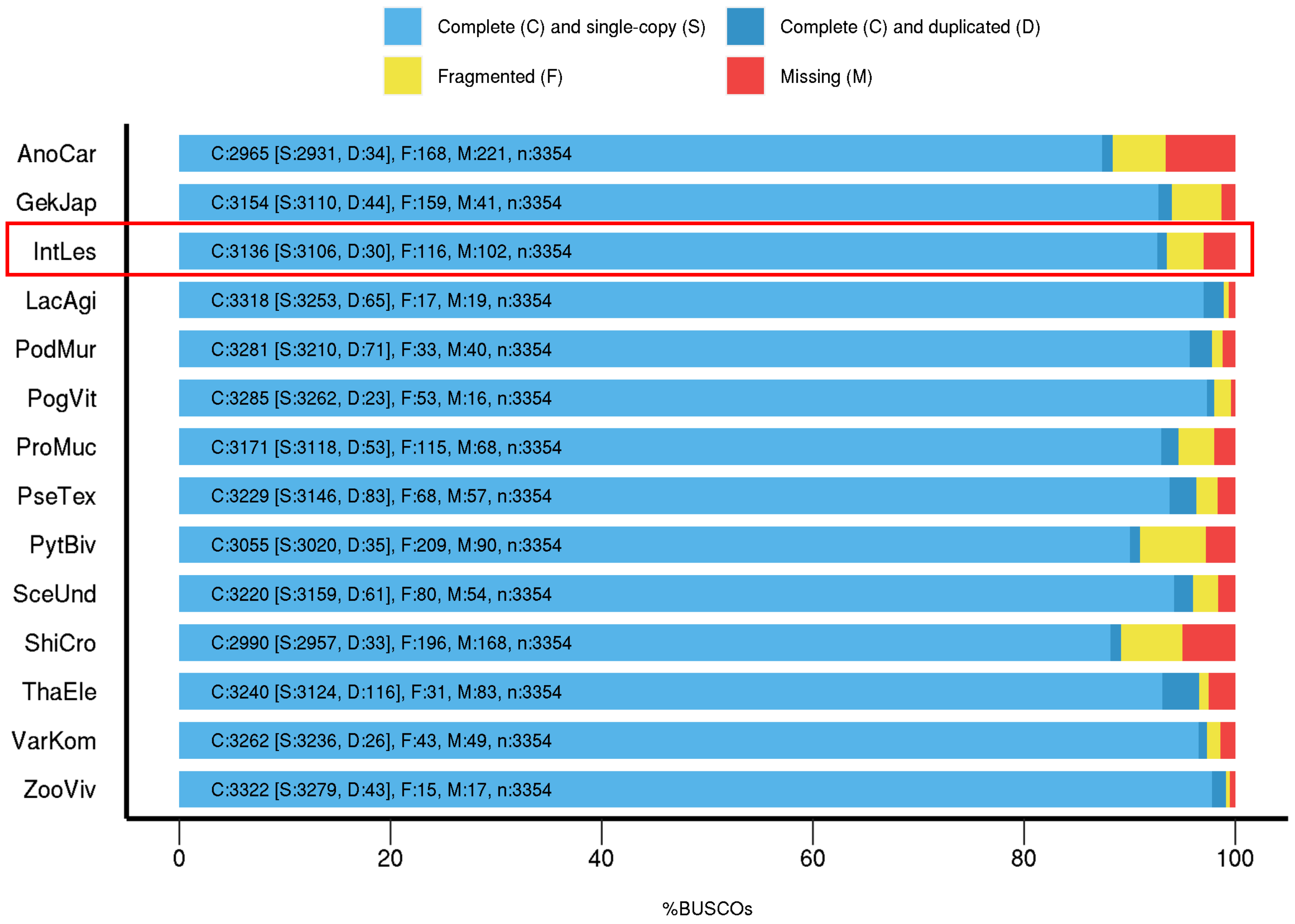

Supplementary Figure S5. BUSCO analysis of the predicted protein coding sequences from publicly available Squamate genomes listed in Supplementary Table S2. AnoCar; *Anolis carolinensis*, GekJap; *Gekko japonicus*, IntLes; *Intellagama lesueurii*, LacAgi; *Lacerta agilis*, PodMur; *Podarcis muralis*, PogVit; *Pogona vitticeps*, ProMuc; *Protobothrops mucrosquamatus*, PseTex; *Pseudonaja textilis*, PytBiv; *Python bivittatus*, SceUnd; *Sceloporus undulatus*, ShiCro; *Shinisaurus crocodilurus*, ThaEle; *Thamnophis elegans*, VarKom; *Varanus komodoensis*, ZooViv; *Zootoca vivipara*.

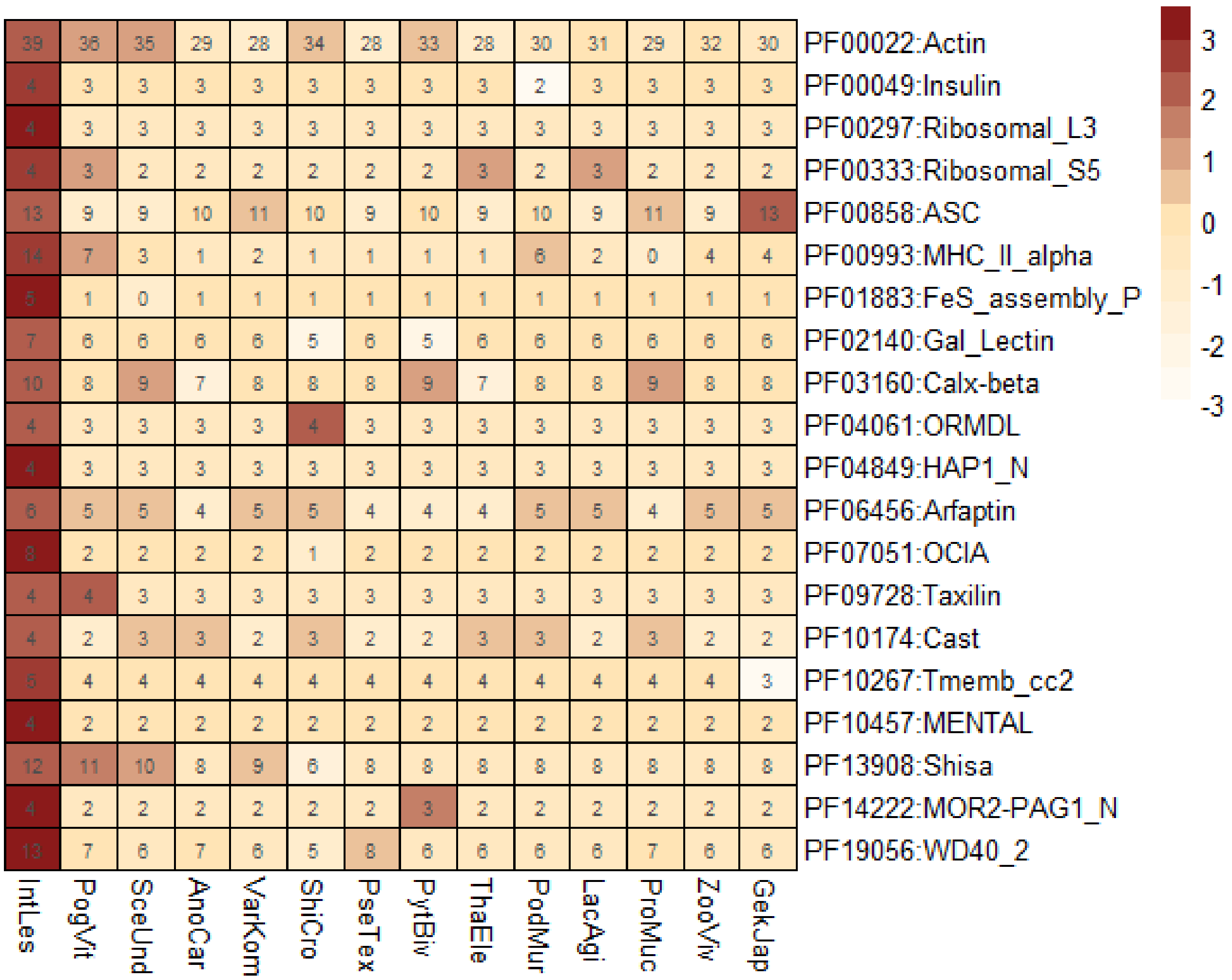

Supplementary Figure S6. Expanded protein family domains in *I. lesueurii* when compared with 13 other squamate gene sets. Scale bar represents the row z-score. AnoCar; *Anolis carolinensis*, GekJap; *Gekko japonicus*, IntLes; *Intellagama lesueurii*, LacAgi; *Lacerta agilis*, PodMur; *Podarcis muralis*, PogVit; *Pogona vitticeps*, ProMuc; *Protobothrops mucrosquamatus*, PseTex; *Pseudonaja textilis*, PytBiv; *Python bivittatus*, SceUnd; *Sceloporus undulatus*, ShiCro; *Shinisaurus crocodilurus*, ThaEle; *Thamnophis elegans*, VarKom; *Varanus komodoensis*, ZooViv; *Zootoca vivipara*.

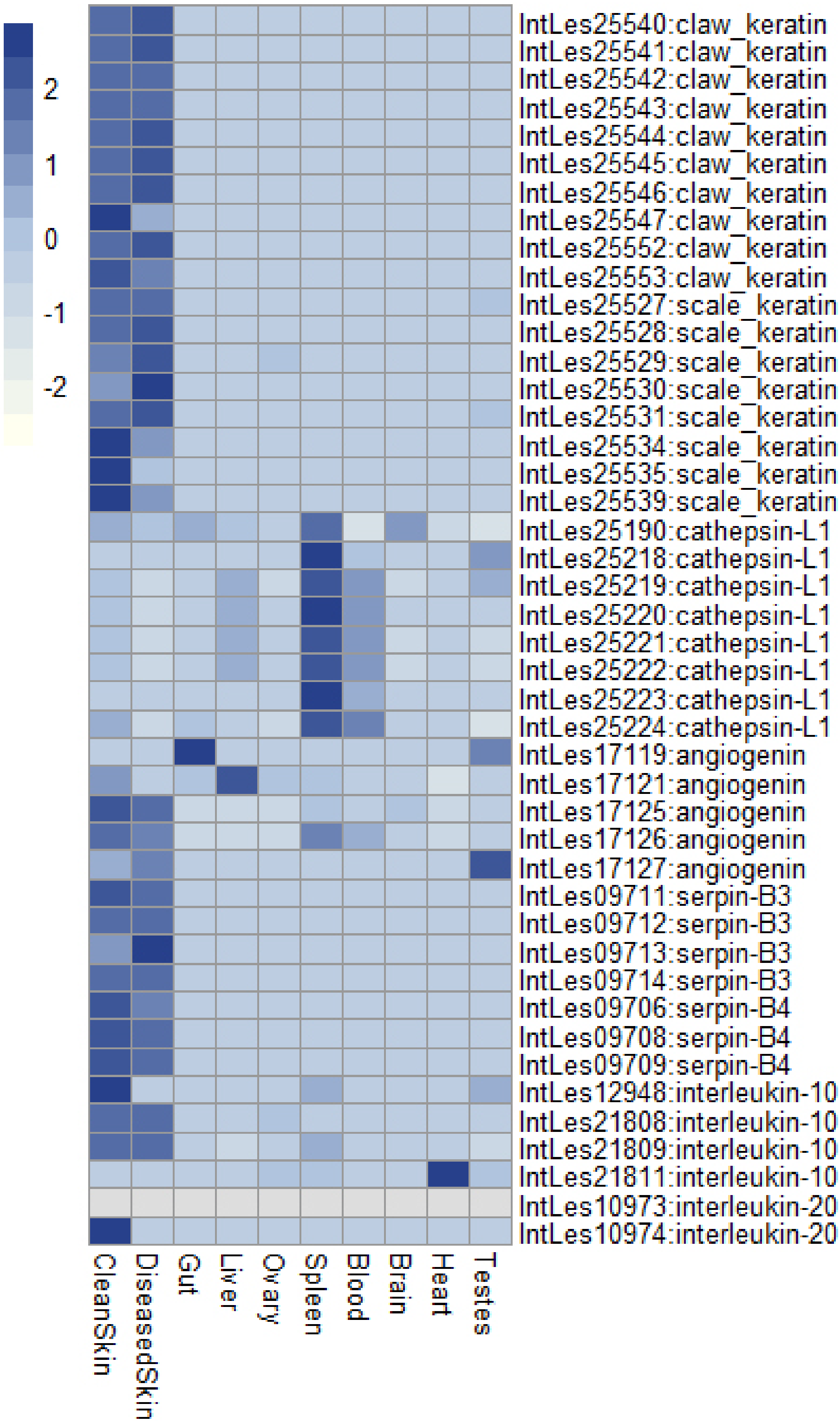

Supplementary Figure S7. The tissue-based expression of the expanded orthologous gene groups in *I. lesueurii* that were identified to be linked to inflammation and wound healing. These gene groups are clearly expressed more highly in the skin and spleen. Scale bar represents the row z-score.

Supplementary Tables

Supplementary Table S1. Classification and number of the repetitive elements identified in the *Intellagama* genome assembly.

Element type	Number of elements	Length occupied	Percentage of sequence
Retroelements	1,022,891	340,859,495 bp	18.75%
SINEs:	269,020	37,814,311 bp	2.08%
Penelope	47,113	9,436,072 bp	0.52%
LINEs:	665,093	235,386,876 bp	12.95%
L2/CR1/Rex	354,205	108,672,390 bp	5.98%
R1/LOA/Jockey	184	14,484 bp	0.00%
R2/R4/NeSL	16,108	6,239,541 bp	0.34%
RTE/Bov-B	204,238	87,113,724 bp	4.79%
L1/CIN4	31,516	20,870,616 bp	1.15%
LTR elements:	88,778	67,658,308 bp	3.72%
BEL/Pao	1,155	60,498 bp	0.00%
Ty1/Copia	5,800	4,411,099 bp	0.24%
Gypsy/DIRS1	45,750	43,573,357 bp	2.40%
Retroviral	23,000	4,520,037 bp	0.25%
DNA transposons	231,009	43,929,660 bp	2.42%
hobo-Activator	125,769	22,624,842 bp	1.24%
Tc1-IS630-Pogo	60,340	17,433,175 bp	0.96%
PiggyBac	690	149,604 bp	0.01%
Tourist/Harbinger	5,497	335,292 bp	0.02%
Other (Mirage, P-element, Transib)	99	4,310 bp	0.00%
Rolling-circles:	3,828	240,229 bp	0.01%
Unclassified:	1,459,168	311,810,615 bp	17.15%
Total interspersed repeats:		696,599,770 bp	38.32%
Small RNA:	5,696	737,934 bp	0.04%
Satellites:	6,520	774,116 bp	0.04%
Simple repeats:	409,407	24,089,903 bp	1.33%
Low complexity:	44,685	9,309,377 bp	0.51%

* most repeats fragmented by insertions or deletions have been counted as one element

Supplementary Table S2. Summary statistics and accession numbers of the published genomes included in the comparative analysis performed in this study.

Lepidosauria > Squamata		Taxonomy		Genome Stats			
**Species**	**ID**	**Family**	**Common Name**	**# Prot seqs**	**Scaffold N50**	**Total Size**	**Genbank Accession**
Intellagama lesueurii	**IntLes**	**Agamidae**	**eastern water dragon**	**23,675**	**269.1 Mb**	**1.8Gb**	**GCA_037013535.1**
Pogona vitticeps	PogVit	Agamidae	central bearded dragon	18,971	2.5 Mb	1.7 Gb	GCF_900067755.1
Thamnophis elegans	ThaEle	Colubridae	western terrestrial garter snake	19,034	100.9 Mb	1.7 Gb	GCF_009769535.1
Anolis carolinensis	AnoCar	Dactyloidae	green anole	19,418	150 Mb	1.8 Gb	GCF_000090745.1
Pseudonaja textilis	PseTex	Elapidae	eastern brown snake	19,403	14.7 Mb	1.6 Gb	GCF_900518735.1
Gekko japonicus	GekJap	Gekkonidae	Japanese gecko	19,548	707.7 Kb	2.5 Gb	GCF_001447785.1
Lacerta agilis	LacAgi	Lacertidae	Sand lizard	20,364	86.6 Mb	1.4 Gb	GCF_009819535.1
Podarcis muralis	PodMur	Lacertidae	common wall lizard	21,295	92.4 Mb	1.5 Gb	GCF_004329235.1
Zootoca vivipara	ZooViv	Lacertidae	common lizard	20,345	92.8 Mb	1.5 Gb	GCF_011800845.1
Sceloporus undulatus	SceUnd	Phrynosomatidae	fence lizard	19,946	275.6 Mb	1.9 Gb	GCF_019175285.1
Python bivittatus	PytBiv	Pythonidae	Burmese python	19,892	214 Kb	1.4 Gb	GCF_000186305.1
Shinisaurus crocodilurus	ShiCro	Shinisauridae	Chinese crocodile lizard	20,150	296.9 Mb	2.2 Gb	GCA_021292165.1
Varanus komodoensis	VarKom	Varanidae	Komodo dragon	18,030	23.8 Mb	1.5 Gb	GCF_004798865.1
Protobothrops mucrosquamatus	ProMuc	Viperidae	pit viper	20,607	424.1 Kb	1.7 Gb	GCF_001527695.2
*Pogona vitticeps**	PogVit	Agamidae	central bearded dragon	N/A	257 Mb	1.7 Gb	N/A

*HiC genome for *Pogona vitticeps* is available via DNAZoo https://www.dnazoo.org/assemblies/pogona_vitticeps

Supplementary Table S3. Gene ontology (GO) enrichment of the 223 orthogroups that were identified as unique to the *Intellagama* genome when compared only with the gene sets from *Pogona vitticeps*, *Anolis carolinensis and Sceloporus undulatus.*

GO ID	GO Description	# Clusters	GO Category*	FDR
GO:0006310	DNA recombination	2	BP	0.000169621
GO:0050982	detection of mechanical stimulus	2	BP	0.000236687
GO:0046872	metal ion binding	6	MF	0.000430907
GO:0003677	DNA binding	2	MF	0.002477693
GO:0006955	immune response	2	BP	0.00270471
GO:0005654	nucleoplasm	2	CC	0.002940856
GO:0019236	response to pheromone	2	BP	0.015739164

*BP: Biological Process; MF: Molecular Function; CC: Cellular Component

Supplementary Table S4. List of software used for assembly annotation and analysis.

Software	Version	Reference in main text
Assembly Workflow		
Hifiasm	v0.14-r312	(Cheng et al. 2021)
Juicer	v1.6	(Durand et al. 2016)
3D-DNA	v201008	(Dudchenko et al. 2017)
Juicebox Assembly Tools	v2.20.00	(Dudchenko et al. 2018)
TGS-GapCloser	v1.0.1	(Xu et al. 2020)
BUSCO	v5.1.3	(Manni et al. 2021)
Jellyfish	v2.3.0	(Marçais and Kingsford 2011)
GenomeScope	v2.0	(Ranallo-Benavidez et al. 2020)
Genome annotation		
HiSat2	v2.1.0	(Kim et al. 2015)
SAMtools	v1.5	(Li et al. 2009)
StringTie2	v2.2.0	(Pertea et al. 2016)
RepeatMasker	v4.1.2-p1	(Smit, AFA, Hubley, R & Green 2015)
RepeatModeler	v2.0.1	(Flynn et al. 2020)
Fgenesh++	Softberry, Inc.	(Solovyev et al. 2006)
MAKER	v3.01.03	(Holt and Yandell 2011)
Comparative genomics		
Minimap2	v2.24	(Li 2018)
D-GENIES	v1.4.0	(Cabanettes and Klopp 2018)
Mauve	v1.1.3	(Darling et al. 2010)
Geneious Prime	v2023.2.1	Biomatters Ltd.
OrthoVenn2	2018	(Xu et al. 2019)
OrthoFinder	v2.4.0	(Emms and Kelly 2019)
MAFFT	*Compiled with above*	(Katoh and Standley 2013)
IQ-TREE 2	v2.2.0	(Minh et al. 2020)
HMMER(hmmscan)	v3.1	(Potter et al. 2018)
Pfam A database	release 34.0	(Finn et al. 2014)
CAFE	v4.2.1	(Han et al. 2013)
FastTree	v2.1.12	(Price et al. 2010)
Methylation analysis		
TrimGalore	v0.6.7	https://github.com/FelixKrueger/TrimGalore
NuMetRRBS	Dec 2017	https://github.com/nugentechnologies/NuMetRRBS
Bismark	v0.22.3	(Krueger and Andrews 2011)
methylKit	v 1.20.0	(Akalin et al. 2012)
